# Establishment of a Biorepository for Down Syndrome: Experience of the Inter-Institutional Multidisciplinary BioBank - BioBIM

**DOI:** 10.24976/Discov.Med.202436184.85

**Published:** 2024-05

**Authors:** Claudia Condoluci, Raffaele Palmirotta, Jeanne B. Lawrence, Kelly P. Smith, Anna R. Casini, Gabriella Di Girolamo, Lucia A. Majolini, Maria G. Valente, Antonella Spila, Chiara Miele, Patrizia Ferroni, Fiorella Guadagni

**Affiliations:** 1 Centre for Child Development, IRCCS San Raffaele Pisana, 00166 Rome, Italy; 2 Interdisciplinary Department of Medicine, School of Medicine, University of Bari “Aldo Moro”, 70124 Bari, Italy; 3 Department of Neurology, University of Massachusetts Medical School, Worcester, MA 01655, USA; 4 Department of Neurosciences, San Giovanni-Addolorata Hospital, 00184 Rome, Italy; 5 InterInstitutional Multidisciplinary Biobank (BioBIM), IRCCS San Raffaele, 00166 Rome, Italy; 6 Department for the Promotion of Human Sciences and Quality of Life, San Raffaele Roma Open University, 00166 Rome, Italy

**Keywords:** Down syndrome, biospecimens, biobank, genetics, Alzheimer’s disease, congenital heart disease

## Abstract

**Background:**

Down syndrome, or Trisomy 21, is the leading genetic cause of cognitive disability in children and is associated with a high risk of several comorbidities, particularly congenital heart defects, early onset Alzheimer’s disease, leukaemia, and autoimmune disorders.

**Objective:**

This study describes the design, methods, and operational procedures employed to establish a biobank dedicated to Down syndrome that can support research projects investigating the effects of various genetic and environmental factors on this complex disease.

**Methods:**

Blood was collected from all recruited subjects, processed, aliquoted and immediately frozen at −80 °C in the Interinstitutional Multidisciplinary BioBank (BioBIM) facilities. A small aliquot of the sample was used to perform blood tests for which analysis would not be feasible at a later date, such as blood cell counts. Each biological sample was coded, assigned a Standard PREanalytical Code, and registered in the oloBIOBANK software connected to a medical card containing all the donor’s anamnestic data. All samples were stored under continuous real-time temperature recording using a freezer connected to a T-GUARD alarm system. In addition, a radiofrequency identification tracking system strictly monitored each cryopreservation operation performed throughout the sample lifecycle.

**Results:**

Biological samples were collected from 454 individuals with Down syndrome from 2007 to 2023. A total of 2233 biological samples were available for research purposes, including whole blood in different anticoagulants, serum, plasma, and frozen peripheral blood mononuclear cells. The quality of the nucleic acids obtained through specific standard operating procedures demonstrated that these samples were appropriate for clinical and basic research.

**Conclusion:**

By establishing this biobank, we have gathered a significant number of biological samples and clinical data from individuals with Down syndrome, thereby fostering collaboration between different research groups in an open and transparent manner. Sharing expertise and resources among scientists will ultimately facilitate the transfer of knowledge to clinical practice, leading to the development of more effective therapeutic treatments to improve the outcomes and quality of life of patients with Down syndrome.

## Introduction

### Down Syndrome

Down syndrome (DS) (OMIM #190685) is the most common chromosomal abnormality in live births and the leading cause of cognitive disabilities in children. It was first defined in 1866 by London Hospital physician Langdon Down [1], who described the main features of the syndrome as epicanthal folds, wide face, macroglossia, cognitive disability, and shortened life span. In 1959, Lejeune *et al.* [2] demonstrated that DS was caused by the presence of three copies of chromosome 21, with 95% of cases caused by a free extra chromosome 21 which arises *de novo* [3,4]. About 3–4% of cases involve translocations in which the extra chromosome 21 material is translocated to another chromosome; these unbalanced translocations can be inherited from a normal parent carrying a balanced translocation [4]. Approximately 1% of other cases may be caused by genetic “mosaicism” [5], characterised by the presence of both euploid and trisomic cell populations and clinical presentation is usually attenuated [4,6]. DS occurs in less than 1 out of 700 live births in the USA and affects millions worldwide [4,7,8]. The prevalence in Europe is 10.12 per 10,000 births [9]. In addition to variable levels of cognitive disability, individuals with DS are at a higher risk (~50%) for congenital heart defects and almost inevitable early onset Alzheimer’s disease, as well as an increased risk of autoimmune disorders, leukaemia, autism, and other potential comorbidities [10-13].

The understanding of the biology of DS has greatly increased in recent years; however, the specific cellular impacts and molecular mechanisms responsible for DS-related cognitive impairment, as well as other comorbidities, are yet to be elucidated. DS mouse models have been commonly used for many years; however, recent initiatives have emphasised the need for larger clinical cohorts and genomic-scale resources to study this condition in humans. A high degree of variability among individuals has been described, reflecting the complexity and differences in genetic backgrounds of individuals with trisomy 21 [7,11]. Since 2000, significant results have been achieved in dissecting the genetic content of the long arm of chromosome 21 (*HSA21*), which was fully sequenced and catalogued in an international collaborative study [14]. However, whole-genome studies in large cohorts of clinically characterised individuals with DS have recently become a priority. These offer substantial opportunities to study the molecular pathogenic mechanisms from individual genetic and phenotypic profiles, thereby developing targeted therapeutic interventions. This is important for individuals with DS but is also relevant to their comorbidities that affect the non-DS population such as congenital heart disease and Alzheimer’s disease (AD).

### Design and Development of a Dedicated Biobank

Planning and developing a dedicated biobank is crucial for research focused on genetic mechanisms, potential interactions with environmental factors, and their impact on the diverse phenotypes of individuals with DS [11]. Biological banks are defined by the Oviedo Convention as “operational units that provide a service for the preservation and management of biological material and associated clinical data in accordance with good laboratory practice, privacy law and ethics guidelines”. They constitute a fundamental resource, even many years after sample collection, and are essential prerequisites and fundamental supports for research and clinical trials of rare genetic diseases, especially from the current perspective of personalised medicine [15]. There are few biobanks dedicated to DS worldwide and none in Italy, therefore, our group decided to establish a biorepository dedicated to DS within the Interinstitutional Multidisciplinary BioBank (BioBIM) of the Scientific Institute for Research, Hospitalisation, and Healthcare (IRCCS), San Raffaele, Rome, Italy. BioBIM is a non-profit service unit for translational research in the medical field, configured as a Biological Resource Centre (BRC) and provided with a proprietary Data Lake for data matching, data mining, and integration. Its activities include obtaining, storing, processing, and sharing biological samples and their associated health data. Processing health data includes but is not limited to data storage, pseudo-anonymisation, anonymisation, curation, analysis, data transfer, and data sharing. BioBIM is well-established within the national health system and international research community. This has created a strong network of collaborations with other research institutions and biobanks; ultimately strengthening the possibility of promoting cultural exchange at a national and international level and supporting projects, multicentre protocols, and research agreements. BioBIM is ISO certified and accredited by the Italian Ministry of Health (https://directory.bbmri.it/#/board).

Herein, we present the procedures and pre-analytical, analytical, and cryopreservation processes used in the BioBIM DS biorepository which aims to provide high-quality biological samples and matched clinical-genetic data to the scientific community. The availability of both mutually correlated elements provides an important resource for studying DS and its clinical evolution and developing new diagnostic tests and targeted treatments [16,17]. We are confident that this resource, which features hundreds of well-characterised individuals with DS, will be of great interest and value to the DS research community.

## Materials and Methods

### Recruitment of Individuals with DS

Starting in 2007, individuals with DS included in the project were all admitted to the Paediatric Rehabilitation and Development Disabilities Department; instituted in the early 2000s by Prof. Giorgio Albertini of the IRCCS. This is a highly specialised reference centre that focuses on childhood disabilities, including clinical practice, rehabilitation, and scientific research. The only criterion for enrolment was written informed consent signed by a parent or legal guardian. All subjects with DS for which informed consent was available were enrolled and prospectively followed up in the outpatient clinic for treatment and periodic care. Exclusion criteria included lack of written informed consent, other chromosomal disorders, or incomplete clinical information. For all individuals with DS included in the biobank, complete clinical data were recorded and monitored during follow-up, together with additional sampling whenever possible by a highly specialised team. The diagnosis and management of comorbidities were handled using a multidisciplinary approach that included paediatricians, cardiologists, orthopaedic surgeons, endocrinologists, neurologists, and psychologists. Clinical information was coded according to the International Classification of Diseases, Ninth Revision (ICD-9) [18], a medical classification used worldwide in epidemiology, public health surveillance, health management, and clinical purposes to promote international comparability in the collection, processing, classification, and presentation of diseases.

All participants were evaluated using standardised and Italian-translated versions of the Brunette-Lezine, Griffiths, and Wechsler Preschool and Primary Scale of Intelligence assessments of psychomotor development in preschool children [19-21]. The intelligence quotient was assessed using Raven’s Coloured and Standard Progressive Matrices [22,23]. In addition, the Wechsler Adult Intelligence Scale and age-based Wechsler Intelligence Scale for Children were used [24,25]. Language abilities were evaluated by administering the Italian equivalents of the battery test for language assessment, the Rustioni test for language assessment in Italian, and the Cornoldi test for learning assessment [26-28].

Before participating in the project, all subjects and their parents or legal guardians were adequately informed by a paediatrician about the importance of biobanks for scientific research and the reasons for the collection of biological samples. A printed information sheet was provided to the family, and an informed consent form was signed by both the legal guardians and physicians. All participants were recruited and followed up under appropriate institutional ethics approval and in accordance with the principles of the World Medical Association Declaration of Helsinki.

### Biospecimen Collection

Approximately 10 mL of venous peripheral blood was collected by a single venipuncture over 20–30 s into Vacutainer tubes (BD, Franklin Lakes, NJ, USA) containing different anticoagulants: K2 EDTA (3 mL tube; 367838); Na Citrate (2.7 mL tube; 363095); or SSTTM II Advance tubes Silica (3.5 mL tube; 366127). When feasible, an additional blood aliquot was collected into two lithium heparin vacutainer tubes (4 mL tube; 367883) for isolation of peripheral blood mononuclear cells (PBMCs). This method allowed the widest possible range of blood sample derivatives to be stored for future studies. The ambient temperature of the BioBIM building was maintained for all work environments at 18–22 °C. Immediately after collection, the samples were transferred to the BioBIM Laboratory, where they were processed using the most appropriate standard operating procedures [29].

After appropriate separation of plasma and serum by centrifugation, all samples were aliquoted into 1.8 mL cryotubes and immediately frozen at −80 °C [30,31]. A small aliquot of the sample was used to perform blood tests which would not be feasible at a later date such as blood cell counts. Information on the instruments used for routine laboratory tests is presented in Table 1.

**Fig. 1.**
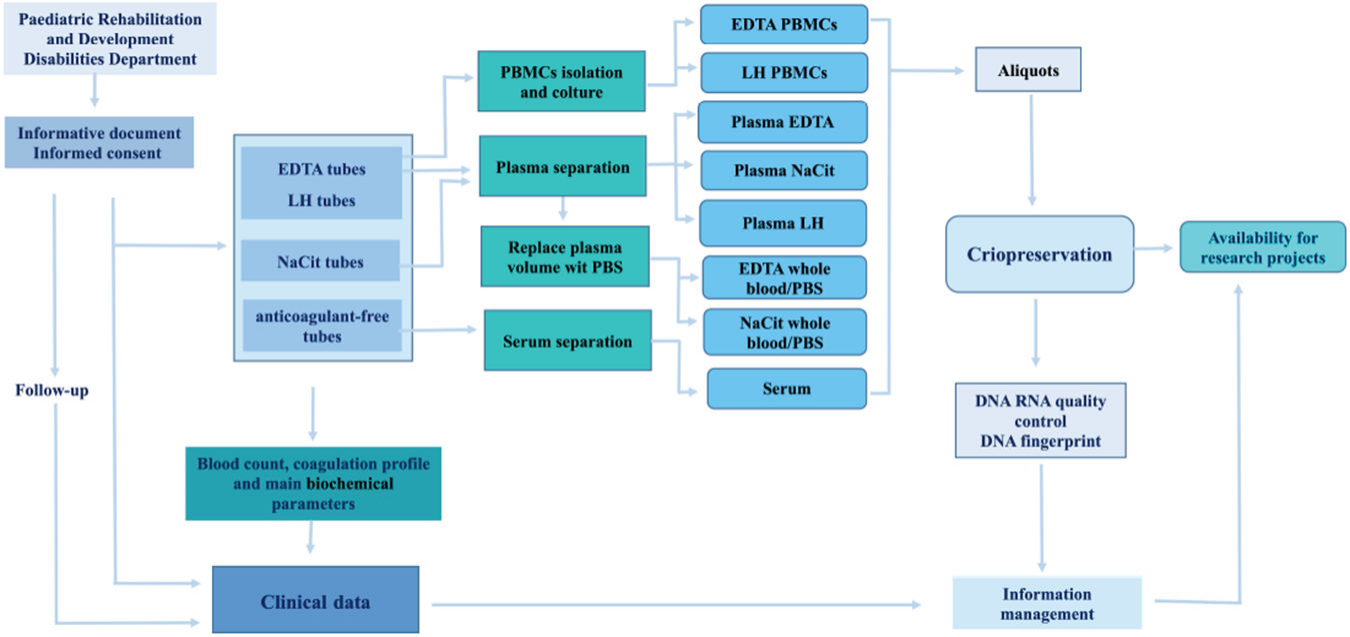
Workflow of biosamples and clinical data acquisition within the general organization of the Down syndrome section of BioBIM, from recruitment of individuals with Down syndrome (DS) to sample cryopreservation (performed with Microsoft^®^ Power Point^®^^©^ 2013 Microsoft Corporation, Redmond, WA, USA). BioBIM, Interinstitutional Multidisciplinary BioBank; PBMCs, peripheral blood mononuclear cells.

The sample processing workflow is shown in Fig. 1. PBMCs were isolated from blood from EDTA or lithium heparin collection tubes using phosphate-buffered saline and Lympholyte^®^-H (CL5010, Cedarlane, Hornby, ON, Canada; CL5010) before resuspending in 1 mL of denaturing solution and storing 1–3 aliquots at −80 °C [32,33]. In addition, the viable PBMCs required for immunopheno-typing and functional assays, such as chromosome-based analysis, detection of genotoxicity, or cytotoxicity assays, were processed as previously described and 1–3 vials of each stored in liquid nitrogen [33,34]

All freezers were monitored, continuous real-time temperature recorded, and fitted with a T-GUARD alarm system (Biomed-Consulting, Milan, Italy).

### Information Management

One of the most crucial aspects of setting up a biobank is the implementation of automated processes for the integrated traceability of samples and their storage and faster sample retrieval; improving sample integrity, traceability, and safety and increasing efficiency.

To this end, each biological sample was coded and registered in the oloBIOBANK software (https://www.olomedia.com/biobank-biobanca/) (Olomedia, Palermo, Italy). This is a web-based system that provides a host server that stores donor data and information on samples and aliquots. The oloBIOBANK software allows the management of individual sample preservation and traceability, regardless of the storage method or location, by connecting it to a donor card which represents a medical record containing all the donor’s anamnestic data.

oloBIOBANK uses the Standard PREanalytical Code (SPREC) designed by the International Society for Biological Environment Repositories to identify samples [35-37]. This assigns a 7-element code to each sample corresponding to seven pre-analytical variables, with different strings of letters for fluids or solid tissues.

BioBIM was validated and a radio-frequency identification (RFID) tracking system was introduced. This is a data identification and storage technology that allows the tracing of the specific paths of each biological sample until freezing. The RFID system designed and developed at the biobank consists of a device that provides a chronological report of the processes involved in each cryopreservation operation, such as door opening/closing, rack insertion/extraction, sample insertion, and the identification of the laboratory operator [29,38].

### Nucleic Acid Extraction Quality Control

Pre-analytical variables, including storage time and ambient temperature, may influence the quality and quantity of the isolated nucleic acids. Considering the large number of samples collected, optimising standard procedures for storing whole blood prior to DNA extraction is a crucial step in a biological repository.

Quality assessment of the cryopreserved biological samples was performed by extracting nucleic acids from randomly selected samples in accordance with the planned timelines. Standard operating procedures from pre-analytical steps to DNA and RNA extraction were previously validated and described [30,32].

Briefly, DNA was extracted from whole blood using the QIAamp DNA Blood Mini Kit (51104, QIAGEN, Hilden, Germany), and RNA was extracted from PBMCs using the QIAamp RNA Blood Mini Kit (52304, QIAGEN, Hilden, Germany). The concentration and quality of the DNA and RNA were assessed using a Nanodrop ND-1000 spectrophotometer (Thermo Fisher Scientific, Waltham, MA, USA). DNA integrity was assessed by PCR to obtain DNA fragments of various lengths, whereas RNA integrity was determined using an Agilent 2100 Bio-analyzer with RNA 6000 Nano and RNA 6000 Pico assays (Agilent Technologies, Santa Clara, CA, USA) [30,32].

Finally, a low-cost and easy-to-use protocol for DNA fingerprinting capable of ensuring DNA identity in a biorepository was validated and introduced within the biobank standard operating procedures [39].

## Results

### Clinical Database

**Fig. 2.**
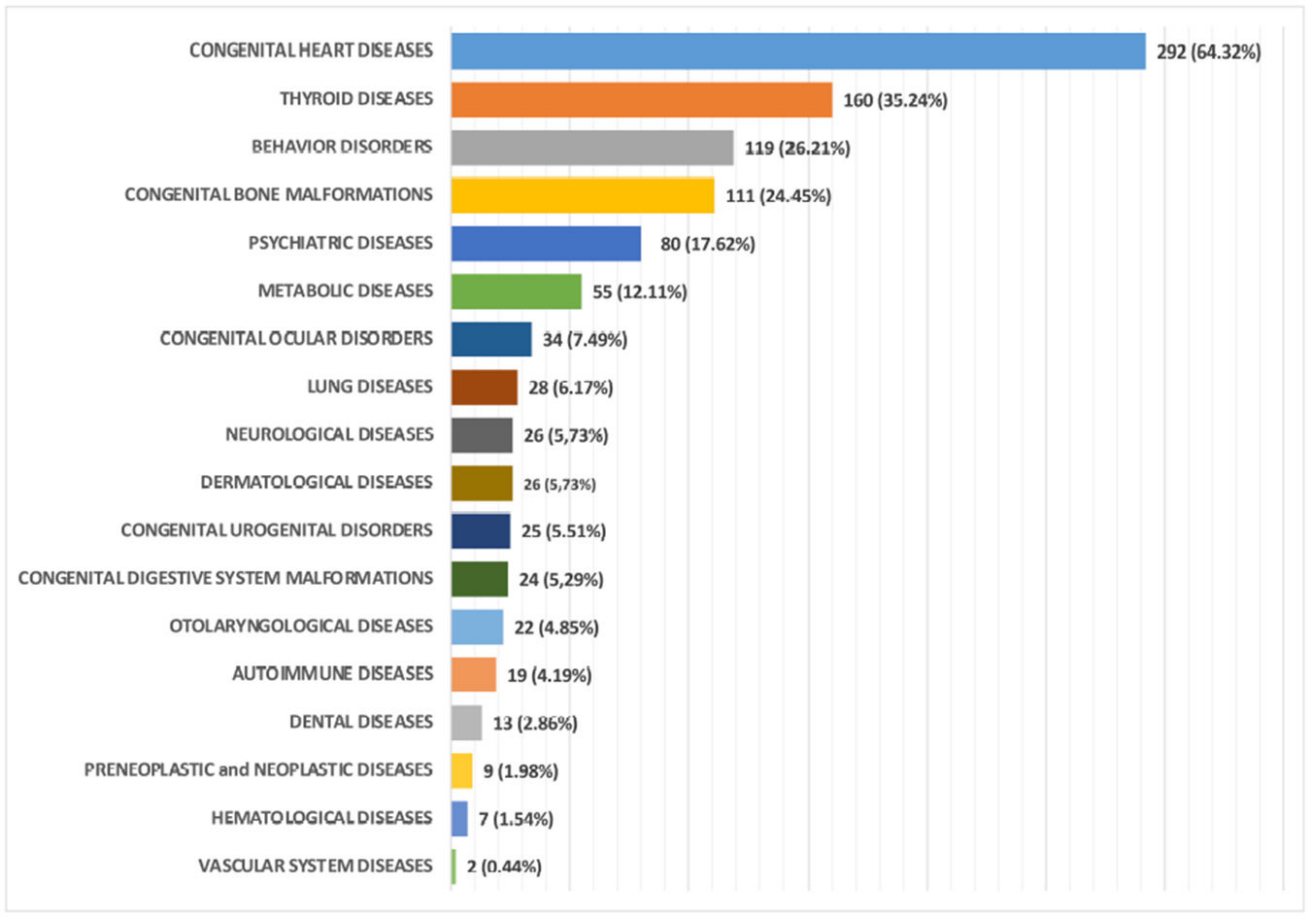
Graphic representation of the incidence of specific comorbidities categorized according to specific disease groups in the 454 Down syndrome afferent to BioBIM. At the end of the bars is the number of instances the type of comorbidity was diagnosed in total and the parentheses indicate the frequency of the presence of the specific condition in the 454 subjects.

**Fig. 3.**
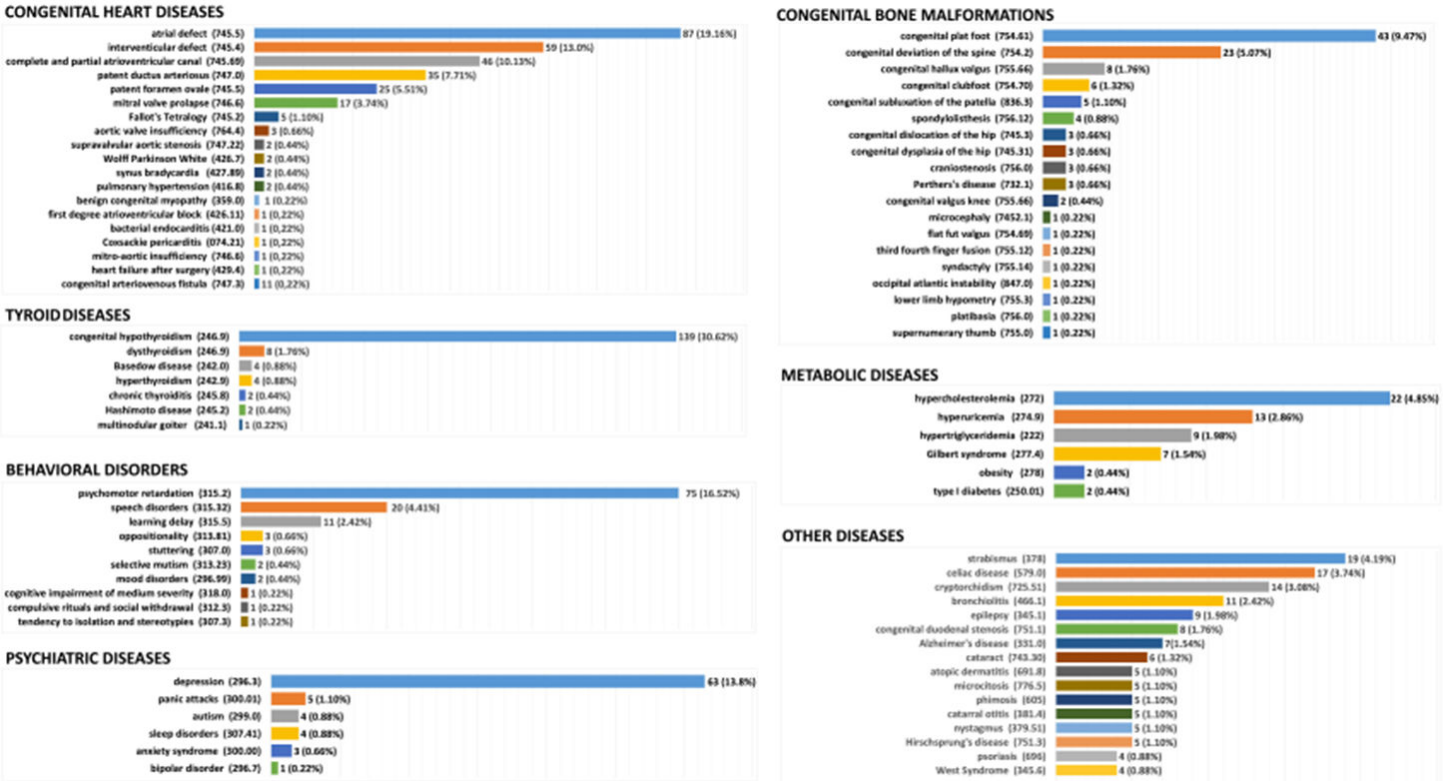
Graphic representation of comorbidities associated with the 454 individuals with Down syndrome in BioBIM. For each disease group, the specific disease and the code according to the International Classification of Diseases, Ninth Revision (ICD-9) [18] is shown on the left, and the right of the bars shows the absolute number and frequency.

To date, a total of 454 subjects, 253 males (55.73%) and 201 females (44.27%) with an average age of 7 years, ranging from 1–55 years, have been enrolled. The majority of participants were referred to our centre from southern Italy (51.10 %) and central Italy (44.05 %); only 3.96% came from Northern Italy and 0.88% from other areas. The chromosomal assessment found a non-disjunction in 418 (92.07%), translocation in 11 (2.42%), and mosaicism in 5 (1.10%) individuals with DS, however, the result of the cytogenetic analysis was not available for 20 (4.41%) cases. Table 2 provides the basic demographic information and age distribution of the enrolled individuals with DS. A summary of the various patient comorbidities associated with the syndrome is shown in Fig. 2, and additional details on the phenotypic subtypes are listed in Fig. 3 (Ref. [18]) and Supplementary Table 1. For each case, all diagnosed conditions were coded using the International Classification of Diseases, Ninth Revision (ICD-9) [18].

As summarised in Table 3, 206 of 454 individuals with DS (45.4%) had congenital heart defects, with a slightly higher percentage in males (52.9%) than in females (47.1%). This was the most common comorbidity and was consistent with previous evidence. Some individuals with DS had more than one diagnosed heart defect, therefore, 292 heart defects were observed, which corresponded to 64.32% of these comorbidities in the 454 subjects. (Fig. 2, Supplementary Table 1). As shown in Fig. 3, atrial defects (ICD-9 745.5), intraventricular defects (ICD-9 745.4), and complete and partial atrioventricular canals (ICD-9 745.7) were the most common heart defects and were detected in 87 (19.16%), 59 (13.0%), and 46 (10.13%) patients, respectively. Other defects observed were 35 (7.71%) patent ductus arteriosus (ICD-9 747.0), 25 (5.51%) patent foramen ovale (ICD-9 745.5), and 17 (3.74%) mitral valve prolapses (ICD-9 746.6). In addition, five (1.10%) patients with tetralogy of Fallot (ICD-9 745.2) and three (0.66%) patients with aortic valve insufficiency (ICD-9 764.4) were included. Other diseases (15 [5.1%]) showed individual frequencies of less than 1% (Fig. 3, Supplementary Table 1).

Various forms of thyroid disease were diagnosed in 160 (35.24%) individuals. Congenital hypothyroidism (ICD-9 243) was by far the most common, representing 139 cases (30.62%), whereas only eight cases of dysthyroidism (ICD-9 246.9) (1.76%) and four cases (0.88%) with both hyperthyroidism (ICD-9 242.9) and Basedow disease (ICD-9 242.0) were identified (Fig. 3, Supplementary Table 1).

In view of the underlying diagnosis of DS, which includes the presence of global developmental delay, only cases in which the delay was greater than “mild” on the specific scale are reported in Supplementary Table 1 and Fig. 3. Psychomotor retardation (ICD-9 315.2), speech disorders (ICD-9 315.32), and learning delay (ICD-9 315.5) were the most frequent disorders, with 75 (16.52%), 20 (4.41%), and 11 (2.42%) cases, respectively, from a total of 119 (26.21%) individuals with behavioural disorders.

Individuals with DS were also evaluated for the presence of bone malformations, which were identified in 111 (24.25%) cases, of which the most frequent pathologies were congenital plantar foot (ICD-9 754.61) in 43 (38.74%) and congenital deviation of the spine (ICD-9 754.2) in 23 (20.72%) individuals (Fig. 3, Supplementary Table 1).

Of the 454 individuals with DS that were recruited, 80 (17.62%) were categorised as having psychiatric disorders. Notably, this percentage was mostly attributable to 63 individuals (78.75%) with a diagnosis of depression (ICD-9 396.3) (Fig. 3, Supplementary Table 1). Older individuals with DS had a general increase in the frequency of depression.

Other metabolic conditions were diagnosed in 55 (12.11%) of the 454 individuals with DS. Hypercholesterolaemia (ICD-9 272), hyperuricaemia (ICD-9 274.9), hypertriglyceridaemia (ICD-9 222), and Gilbert syndrome (ICD-9 277.4) were found in 22 (4.85%), 13 (2.86%), 9 (1.98%), and 7 (1.54%) patients, respectively (Fig. 3, Supplementary Table 1).

Other clinical features such as congenital eye, urogenital and digestive system, lung, dermatologic, otolaryngological, autoimmune, neoplastic, vascular system, and neurological diseases were present in 235 subjects, with a frequency ranging of 0.44–7.49% (Fig. 3, Supplementary Table 1).

Since individuals with DS are now typically raised in families and live longer, we have unfortunately learned that trisomy 21 causes an extremely high frequency (approximately 80%) of early-onset AD. Since the 454 individuals represented in BioBank were mostly children or younger than the age at which AD manifests, the AD frequency in our population was lower than expected. Only seven (1.54%) cases of AD (ICD-9 331.0) were included among the neurological comorbidities; strikingly, these were diagnosed at ages 31, 32, 34, 36, 37 (n = 2), and 46 years. Therefore, of the 47 individuals with DS over the age of 30, with the oldest being 55, seven have been identified with AD and most have been diagnosed at a younger age than expected. Of the 15 cases of age 40 or older, only one was diagnosed with AD at the time of recruitment to the biobank, highlighting the importance of following individuals longitudinally whenever possible.

### Sample Resource

To date, 2233 samples from the 454 individuals with DS enrolled into the biobank have been cryopreserved relative to the time of recruitment, including 454 whole blood samples in EDTA; 428 serum samples; 370 plasma citrated samples, 370 plasma EDTA samples, 370 plasma lithium heparin samples and 241 frozen PBMCs. In multiple instances, the collection of biological samples was repeated during clinical follow-up, on average every three years. Sampling for the second, third, fourth, and fifth times has been performed for 154, 56, 18, 5, and 1 subjects, respectively. There are 850 repeated samples, including 218 whole blood samples; 189 serum samples; 375 plasma samples, citrated, EDTA, and lithium heparin; 46 PBMCs; and 22 urine samples.

An additional 69 samples of whole blood, EDTA and citrated; serum; plasma, citrated, EDTA, and lithium heparin; and live PBMCs obtained from EDTA- and lithium heparin-anticoagulated blood (maintained in liquid nitrogen) were collected from members of the 23 families with DS recruited to the biobank, resulting in a total of 552 biological samples.

The frozen PBMCs had an average cell count of 11.8 × 10^6^/mL, with a range of 4.37–19.95 × 10^6^/mL. Quality checks of nucleic acid extractions indicated that DNA concentrations averaged 35 ng/μL, with a range of 15–5050 ng/μL, and an acceptable absorbance ratio at 260 and 280 nm. Similarly, RNA extraction had an average concentration of 60 ng/μL, with a range of 40–170 ng/μL, and an absorbance ratio at 260/280 nm and 260/230 always higher than 1.9 and 1.8, respectively [30,31].

## Discussion

In this study, we report the creation of a DS biobank within the BioBIM BRC that can provide a freely accessible resource for translational research studies on this population. Our focus on this condition stems from the large number of patients with disabilities who visit our centre, including individuals with DS, and the strong commitment of the IRCCS; whose institutional mission is to guarantee a high-quality and specialised integrated assistance and rehabilitation network. To the best of our knowledge, this is the first dedicated DS biobank in Italy. An advantage of this biobank is its coexistence with a highly specialised laboratory, which has made it possible to carry out verification and analytical tests on the samples to establish the characteristics and criteria for collection and preservation, as demonstrated by scientific papers published as part of the project [29-32,37]. To date, BioBIM has recruited 454 and 80 individuals with DS and AD, respectively. Each biological sample was linked to the detailed sociodemographic and clinical characteristics of the donor.

For some years, several authors and research agencies have promoted the need to establish biological banks dedicated to DS, recognising its importance and that the number of individuals with DS has increased [17,40-42]. Many women over 35 years now experience pregnancies, which significantly increases the risk of DS. Despite the wide availability, they do not always undergo prenatal tests, or after a positive test, choose to continue the pregnancy [43].

Furthermore, owing to better healthcare and preventive medicine, as well as improved socioeconomic integration of individuals with DS, their life expectancy is progressively increasing [17,34]. Recent data indicate that individuals with DS now have a life expectancy of more than 55 years compared to only 25 years in the 1980s [44]. This evolution creates new and more demanding challenges in the field of healthcare for individuals with DS and overcomes the “culture of intractability”, which has characterised this disorder for years [15,41]. A longer life expectancy has increased the need to understand the causes of various comorbidities and their consequent diagnostic, prognostic, and therapeutic implications. An important example is the association between the aging of this population and a greater risk of developing dementia similar to AD, about which little is known and whose diagnosis is particularly challenging [11,13,14,17].

Recently, the LuMind IDSC Foundation and the National Down Syndrome Society, two leading DS research and advocacy organisations in the USA, formulated recommendations to the National Institutes of Health for a research strategy focused on the quality of life and care priorities for individuals with DS by 2030. As part of this health programming plan, which focuses on medical research as a tool to improve the health of individuals with DS, one of the overall priority recommendations was to establish specific centralised DS biobanks [44,45].

In this context, a dedicated biobank with serum, plasma, nucleic acids, and PBMCs linked to continually updated clinical data is the best strategy for conducting translational research studies to identify novel therapeutic approaches for individuals with DS [17]. A biobank is essential for the rapid application of “omics” scientific tools to translational and clinical trials to identify new biomarkers for therapeutic targets and response predictivity [46], guide the decision making process, and optimize the therapeutic strategy; as suggested by the “precision medicine” of DS [15,17].

## Conclusion

The establishment of a biobank dedicated to DS within the BioBIM BRC aims to enhance and develop scientific research by studying different types of biological samples that can be stored and made available for high-quality research projects aimed at improving knowledge about this syndrome. All these samples allow us to study various aspects of the disease, such as genetic, molecular, biochemical, and cellular characteristics, and compare individuals with DS who show substantially different outcomes, particularly comorbidities. Individuals with trisomy 21 may also be compared to euploid controls to better understand the pathogenetic aspects of co-occurring conditions that impact populations with and without DS and identify new therapeutic options.

## Supplementary Material

supplement

## Figures and Tables

**Table 1.** Instruments and manufacturer information for the blood test.

Blood test	Instrument	Manufacturer
Routine chemistry	ARCHITECT c8000	Abbott Laboratories, Abbott Park, IL, USA
Complete and differential blood cell counts	Coulter LH 750 Hematology Analyzer	Beckman Coulter, Brea, CA, USA
Routine coagulation tests	ACL TOP automated coagulometer	Instrumentation Laboratory (IL) Co., Lexington, MA, USA

**Table 2.** Demographic information and age distribution of individuals with Down syndrome included in BioBIM (N = 454).

	Cases number	%
Age group, years*		
1–3	22	4.85
4–6	24	5.29
7–9	42	9.25
10–12	61	13.44
13–15	57	12.56
16–18	63	13.88
19–21	40	8.81
22–24	44	9.69
25–27	30	6.61
28–30	24	5.29
31–33	15	3.30
34–36	11	2.42
37–39	6	1.32
41–43	7	1.54
46–50	5	1.10
51–55	3	0.66
Gender		
Female	201	44.27
Male	253	55.73
Chromosomal assessment		
Non-disjunction	418	92.07
Translocation	11	2.42
Mosaicism	5	1.10
NA	20	4.41
Geographical origin		
Southern Italy	232	51.10
Central Italy	200	44.05
Northern Italy	18	3.96
Other	4	0.88

* Age at the time of recruitment to the biobank.

**Table 3.** Demographic information and age distribution of congenital heart defects in individuals with Down syndrome included in the BioBIM (n = 206).

	Cases number	%
Age group, years*		
1–3	12	5.83
4–6	17	8.25
7–9	24	11.65
10–12	39	18.93
13–15	25	12.14
16–18	28	13.59
19–21	20	9.71
22–24	12	5.83
25–27	11	5.34
29–31	7	3.40
32–37	6	2.91
41–51	5	2.43
Gender		
Female	97	47.09
Male	109	52.91

* Age at the time of recruitment to the biobank.
