# Supplementary material for: Establishment of a Biorepository for Down Syndrome: Experience of the Inter-Institutional Multidisciplinary BioBank - BioBIM
Source: Discov Med. Author manuscript; Available in PMC 2024 Jul 17. (PMC11254168; doi:10.24976/Discov.Med.202436184.85)
Supplement: supplement [file NIHMS2003425-supplement-supplement.docx]

**Supplementary Table 1**: Main clinical manifestations in subjects with Down Syndrome included in the BioBIM (n = 454).

| **ICD-9** | **Clinical manifestations** | **N°** | **%^a^** | **%^b^** |
| --- | --- | --- | --- | --- |
|  | |  |  |  |
| **Congenital heart diseases** | |  |  |  |
| 745.69 | complete and partial atrioventricular canal | 46 | 15.75 | 10.13 |
| 745.5 | atrial defect | 87 | 29.79 | 19.16 |
| 745.4 | interventricular defect | 59 | 20.21 | 13.00 |
| 746.6 | mitral valve prolapse | 17 | 5.82 | 3.74 |
| 745.5 | patent foramen ovale | 25 | 8.56 | 5.51 |
| 747.0 | patent ductus arteriosus | 35 | 11.99 | 7.71 |
| 745.2 | Fallot's Tetralogy | 5 | 1.71 | 1.10 |
| 747.22 | supravalvular aortic stenosis | 2 | 0.69 | 0.44 |
| 426.7 | Wolff Parkinson White | 2 | 0.69 | 0.44 |
| 764.4 | aortic valve insufficiency | 3 | 1.03 | 0.66 |
| 359.0 | benign congenital myopathy | 1 | 0.34 | 0.22 |
| 426.11 | first degree atrioventricular block | 1 | 0.34 | 0.22 |
| 427.89 | synus bradycardia | 2 | 0.69 | 0.44 |
| 421.0 | bacterial endocarditis | 1 | 0.34 | 0.22 |
| 074.21 | Coxsackie pericarditis | 1 | 0.34 | 0.22 |
| 746.6 | mitro-aortic insufficiency | 1 | 0.34 | 0.22 |
| 416.8 | pulmonary hypertension | 2 | 0.69 | 0.44 |
| 429.4 | heart failure after surgery | 1 | 0.34 | 0.22 |
| 747.3 | congenital arteriovenous fistula | 1 | 0.34 | 0.22 |
|  | Total | 292 | 100.0 | 64.32 |
| **Thyroid diseases** | |  |  |  |
| 243 | congenital hypothyroidism | 139 | 86.87 | 30.62 |
| 242.0 | Basedow disease | 4 | 2.50 | 0.88 |
| 242.9 | hyperthyroidism | 4 | 2.50 | 0.88 |
| 246.9 | dysthyroidism | 8 | 5.00 | 1.76 |
| 245.8 | chronic thyroiditis | 2 | 1.25 | 0.44 |
| 241.1 | multinodular goiter | 1 | 0.63 | 0.22 |
| 245.2 | Hashimoto disease | 2 | 1.25 | 0.44 |
|  | Total | 160 | 100.0 | 35.24 |
| **Neurodevelopmental disorders** | |  |  |  |
| 315.2 | psychomotor retardation | 75 | 63.03 | 16.52 |
| 315.32 | speech disorders | 20 | 16.81 | 4.41 |
| 313.81 | oppositionality | 3 | 2.52 | 0.66 |
| 307.0 | stuttering | 3 | 2.52 | 0.66 |
| 313.23 | selective mutism | 2 | 1.68 | 0.44 |
| 315.5 | learning delay | 11 | 9.24 | 2.42 |
| 296.99 | mood disorders | 2 | 1.68 | 0.44 |
| 318.0 | cognitive impairment of medium severity | 1 | 0.84 | 0.22 |
| 312.3 | compulsive rituals and social withdrawal | 1 | 0.84 | 0.22 |
| 307.3 | tendency to isolation and stereotypies | 1 | 0.84 | 0.22 |
|  | Total | 119 | 100.0 | 26.21 |
| **Congenital bone malformations** | |  |  |  |
| 754.61 | congenital plat foot | 43 | 38.74 | 9.47 |
| 754.2 | congenital deviation of the spine | 23 | 20.72 | 5.07 |
| 7452.1 | microcephaly | 1 | 0.90 | 0.22 |
| 754.69 | flat fut valgus | 1 | 0.90 | 0.22 |
| 755.66 | congenital hallux valgus | 8 | 7.21 | 1.76 |
| 754.70 | congenital clubfoot | 6 | 5.42 | 1.32 |
| 745.3 | congenital dislocation of the hip | 3 | 2.70 | 0.66 |
| 745.31 | congenital dysplasia of the hip | 3 | 2.70 | 0.66 |
| 836.3 | congenital subluxation of the patella | 5 | 4.51 | 1.10 |
| 755.66 | congenital valgus knee | 2 | 1.80 | 0.44 |
| 755.12 | third fourth finger fusion | 1 | 0.90 | 0.22 |
| 755.14 | syndactyly | 1 | 0.90 | 0.22 |
| 756.0 | craniostenosis | 3 | 2.70 | 0.66 |
| 732.1 | Perthers's disease | 3 | 2.70 | 0.66 |
| 847.0 | occipital atlantic instability | 1 | 0.90 | 0.22 |
| 756.12 | spondylolisthesis | 4 | 3.60 | 0.88 |
| 755.3 | lower limb hypometry | 1 | 0.90 | 0.22 |
| 756.0 | platibasia | 1 | 0.90 | 0.22 |
| 755.0 | supernumerary thumb | 1 | 0.90 | 0.22 |
|  | Total | 111 | 100.0 | 24.45 |
| **Psychiatric diseases** | |  |  |  |
| 296.3 | depression | 63 | 78.75 | 13.88 |
| 299.0 | autism | 4 | 5.00 | 0.88 |
| 296.7 | bipolar disorder | 1 | 1.25 | 0.22 |
| 300.00 | anxiety syndrome | 3 | 3.75 | 0.66 |
| 300.01 | panic attacks | 5 | 6.25 | 1.10 |
| 307.41 | sleep disorders | 4 | 5.00 | 0.88 |
|  | Total | 80 | 100.0 | 17.62 |
| **Metabolic diseases** | |  |  |  |
| 272 | hypercholesterolemia | 22 | 40.00 | 4.85 |
| 222 | hypertriglyceridemia | 9 | 16.36 | 1.98 |
| 274.9 | hyperuricemia | 13 | 23.63 | 2.86 |
| 277.4 | Gilbert syndrome | 7 | 12.73 | 1.54 |
| 278 | obesity | 2 | 3.64 | 0.44 |
| 250.01 | type I diabetes | 2 | 3.64 | 0.44 |
|  | Total | 55 | 100.0 | 12.11 |
| **Congenital eye diseases** | |  |  |  |
| 378 | strabismus | 19 | 55.88 | 4.19 |
| 743.30 | cataract | 6 | 17.65 | 1.32 |
| 379.51 | nystagmus | 5 | 14.71 | 1.10 |
| 371.6 | keratoconus | 2 | 5.88 | 0.44 |
| 743.6 | tear duct stenosis | 1 | 2.94 | 0.22 |
| 367.0 | astigmatism | 1 | 2.94 | 0.22 |
|  | Total | 34 | 100.0 | 7.49 |
| **Lung diseases** | |  |  |  |
| 514 | bronchopolmonites | 3 | 10.71 | 0.66 |
| 466.1 | bronchiolitis | 11 | 39.29 | 2.42 |
| 486 | pneumonia | 4 | 14.29 | 0.88 |
| 748.61 | bronchiectasis | 1 | 3.57 | 0.22 |
| 490 | bronchitis | 8 | 28.57 | 1.76 |
| 491.21 | obstructive bronchitis | 1 | 3.57 | 0.22 |
|  | Total | 28 | 100.0 | 6.17 |
| **Neurological diseases** | |  |  |  |
| 345.1 | epilepsy | 9 | 34.62 | 1.98 |
| 345,6 | West Syndrome | 4 | 15.38 | 0.88 |
| 781.3 | Developmental disturbance of motor coordination | 3 | 11.54 | 0.66 |
| 780.4 | dizziness | 2 | 7.69 | 0.44 |
| 784.0 | migraine | 1 | 3.85 | 0.22 |
| 331.0 | Alzheimer's disease | 7 | 26.92 | 1.54 |
|  | Total | 26 | 100.00 | 5.73 |
| **Dermatologic diseases** | |  |  |  |
| 695.2 | erythema nodosum | 2 | 7.69 | 0.44 |
| 691.8 | atopic dermatitis | 5 | 19.23 | 1.10 |
| 690.12 | seborrheic dermatitis | 2 | 7.69 | 0.44 |
| 705.8 | dyshidrosis | 1 | 3.85 | 0.22 |
| 528.5 | ringworm or angular cheilitis | 1 | 3.85 | 0.22 |
| 704.8 | folliculitis | 2 | 7.69 | 0.44 |
| 110.0 | pityriasis versicolor | 1 | 3.85 | 0.22 |
| 680 | furunculosis | 2 | 7.69 | 0.44 |
| 706.1 | acne | 2 | 7.69 | 0.44 |
| 696 | psoriasis | 4 | 15.38 | 0.88 |
| 701.1 | elastosis perforans serpiginosa EPS | 1 | 3.85 | 0.22 |
| 709.81 | vitiligo | 1 | 3.85 | 0.22 |
| 692.9 | eczema | 1 | 3.85 | 0.22 |
| 757.4 | alopecia | 1 | 3.85 | 0.22 |
|  | Total | 26 | 100.0 | 5.73 |
| **Congenital urogenital diseases** | |  |  |  |
| 725.51 | cryptorchidism | 14 | 56.00 | 3.08 |
| 605 | phimosis | 5 | 20.00 | 1.10 |
| 778.6 | hydrocele | 1 | 4.00 | 0.22 |
| 753.29 | hydronephrosis | 1 | 4.00 | 0.22 |
| 539.89 | pyelectasis | 2 | 8.00 | 0.44 |
| 752.51 | retractable testicle | 1 | 4.00 | 0.22 |
| 753.3 | bilateral renal ectopia | 1 | 4.00 | 0.22 |
|  | Total | 25 | 100.0 | 5.51 |
| **Congenital malformation of the digestive system** | |  |  |  |
| 750.3 | esophageal atresia | 2 | 8.33 | 0.44 |
| 751.0 | diverticulum of Merckel | 2 | 8.33 | 0.44 |
| 751.1 | congenital duodenal stenosis | 8 | 33.33 | 1.76 |
| 751.1 | congenital duodenal atresia | 3 | 12.51 | 0.66 |
| 750.5 | hypertrophic pyloric stenosis | 1 | 4.17 | 0.22 |
| 537.3 | large bowel obstruction | 1 | 4.17 | 0.22 |
| 751.3 | Hirschsprung's disease | 5 | 20.83 | 1.10 |
| 751.2 | congenital anal atresia | 2 | 8.33 | 0.44 |
|  | Total | 24 | 100.0 | 5.29 |
| **Otolaryngological diseases** | |  |  |  |
| 381.4 | catarrhal otitis | 5 | 22.72 | 1.10 |
| V412 | conductive hearing loss | 3 | 13.63 | 0.66 |
| 475.1 | chronic pharyngitis | 1 | 4.55 | 0.22 |
| 474.11 | tonsil hypertrophy | 3 | 13.63 | 0.66 |
| 750.15 | macroglossia | 1 | 4.55 | 0.22 |
| 476.0 | recurrent laryngitis | 1 | 4.55 | 0.22 |
| 748.3 | subglottic stenosis | 1 | 4.55 | 0.22 |
| 748.3 | laryngomalacia | 1 | 4.55 | 0.22 |
| 387 | otosclerosis | 1 | 4.55 | 0.22 |
| 385.30 | cholesteatoma | 1 | 4.55 | 0.22 |
| 474.00 | chronic tonsillitis | 1 | 4.55 | 0.22 |
| 472.0 | chronic rhinitis | 2 | 9.09 | 0.44 |
| 474.12 | adenoid hypertrophy | 1 | 4.55 | 0.22 |
|  | Total | 22 | 100.0 | 4.85 |
| **Autoimmune diseases** | |  |  |  |
| 579.0 | celiac disease | 17 | 89.47 | 3.74 |
| 714.0 | juvenile idiopathic arthritis | 2 | 10.53 | 0.44 |
|  | Total | 19 | 100.00 | 4.19 |
| **Dental diseases** | |  |  |  |
| V41.6 | chewing difficulties | 3 | 23.08 | 0.66 |
| 523 | hypertrophic gingivitis | 2 | 15.38 | 0.44 |
| 306.8 | bruxism | 3 | 23.08 | 0.66 |
| 524 | dental malocclusion | 2 | 15.38 | 0.44 |
| 520 | dental agenesis | 3 | 23.08 | 0.66 |
|  | Total | 13 | 100.0 | 2.86 |
| **Preneoplastic and neoplastic diseases** | |  |  |  |
| 289.6 | begnin erythrocytosis | 1 | 11.11 | 0.22 |
| 776.4 | polycythaemia | 1 | 11.11 | 0.22 |
| 228 | angioma | 2 | 22.23 | 0.44 |
| 478.4 | human papilloma virus | 1 | 11.11 | 0.22 |
| 213.9 | osteoma osteoid | 1 | 11.11 | 0.22 |
| 211.5 | begnin tumors of gallbladder | 1 | 11.11 | 0.22 |
| V10.60 | nonspecific type of leukemia | 1  1 | 11.11 | 0.22 |
| V10.62 | chronic myeloid leukemia | 1 | 11.11 | 0.22 |
|  | Total | 9 | 100.0 | 1.98 |
| **Hematologic diseases** | |  |  |  |
| 776.5 | microcytosis | 5 | 71.42 | 1.10 |
| 288.0 | neutropenia or low neutrophils count | 1 | 14.29 | 0.22 |
| 280 | iron deficiency anaemia | 1 | 14.29 | 0.22 |
|  | Total | 7 | 100.0 | 1.54 |
| **Vascular system diseases** | |  |  |  |
| 779.7 | leukomalacia | 1 | 50.00 | 0.22 |
| 772.9 | intraparenchymal cerebral haemorrhage | 1 | 50.00 | 0.22 |
|  | Total | 2 | 100.0 | 0.44 |

*^a^ Percentage related to the frequency of the disease within the disease group considered.*

*^b^ Percentage referred to the total of cases of Down syndrome (n = 454).*

*ICD-9: the International Classification of Diseases, Ninth Revision.*
